# Current epidemiological status of Middle East respiratory syndrome coronavirus in the world from 1.1.2017 to 17.1.2018: a cross-sectional study

**DOI:** 10.1186/s12879-019-3987-2

**Published:** 2019-04-27

**Authors:** Kazhal Mobaraki, Jamal Ahmadzadeh

**Affiliations:** Epidemiologist in Social Determinants of Health Research Center, Urmia University of Medical Sciences, Resalat Street, Urmia, Iran

**Keywords:** Middle East respiratory syndrome coronavirus, Case fatality rate, Descriptive epidemiology, Disease outbreak, Emerging diseases

## Abstract

**Background:**

Middle East respiratory syndrome coronavirus (MERS-CoV) is considered to be responsible for a new viral epidemic and an emergent threat to global health security. This study describes the current epidemiological status of MERS-CoV in the world.

**Methods:**

Epidemiological analysis was performed on data derived from all MERS-CoV cases recorded in the disease outbreak news on WHO website between 1.1.2017 and 17.1.2018. Demographic and clinical information as well as potential contacts and probable risk factors for mortality were extracted based on laboratory-confirmed MERS-CoV cases.

**Results:**

A total of 229 MERS-CoV cases, including 70 deaths (30.5%), were recorded in the disease outbreak news on world health organization website over the study period. Based on available details in this study, the case fatality rate in both genders was 30.5% (70/229) [32.1% (55/171) for males and 25.8% (15/58) for females]. The disease occurrence was higher among men [171 cases (74.7%)] than women [58 cases (25.3%)]. Variables such as comorbidities and exposure to MERS-CoV cases were significantly associated with mortality in people affected with MERS-CoV infections, and adjusted odds ratio estimates were 2.2 (95% CI: 1.16, 7.03) and 2.3 (95% CI: 1.35, 8.20), respectively. All age groups had an equal chance of mortality.

**Conclusions:**

In today’s “global village”, there is probability of MERS-CoV epidemic at any time and in any place without prior notice. Thus, health systems in all countries should implement better triage systems for potentially imported cases of MERS-CoV to prevent large epidemics.

## Background

Middle East respiratory syndrome coronavirus (MERS-CoV) infection is considered to cause a new viral epidemic [1], and was first reported in a patient who died from a severe respiratory illness in a hospital in Jeddah, Saudi Arabia, in June 2012 [2, 3]. From 1.1.2012 to 17.1.2018, world health organization (WHO) has notified a total of 2143 laboratory-confirmed cases of MERS-CoV, including at least 750 deaths related to this infection from 27 countries around the world [4]. The origin of MERS-CoV has been widely discussed. Initially, a bat reservoir was posited based on phylogenetic similarity of certain bat coronaviruses with MERS-CoV. However, there has been no clear bat source of infection or a consistent history of contact with bats in known cases of MERS-CoV to date [5, 6]. Another source such as dromedary was later introduced as a possible reservoir in some studies [7–10].

Some studies have declared that all cases of MERS-CoV were directly or indirectly linked to residence or travel to 10 countries: Saudi Arabia, UAE, Jordan, Qatar, Kuwait, Oman, Yemen, Egypt, Iran, and Lebanon [6, 11]. The MERS-CoV infection has high mortality rates, especially in patients with comorbidities such as diabetes and renal failure, evoking global concern and intensive discussion in the media along with respiratory droplet route of its transmission [12]. Laboratory-confirmed MERS-CoV cases have been reported during hospital-based cluster outbreaks between 1.1.2017 to 17.1.2018, and cases are still detected throughout the year [4]. The occurrence of a large number of MERS-CoV cases and their associated deaths in the world indicate that this disease must be considered as a severe threat to public health [13] because millions of pilgrims from 184 countries converge in Saudi Arabia each year to perform Hajj and Umrah ceremony. Upon their return to home, pilgrims hold a ceremony attended by family members and friends. Oriental etiquette to share hospitality with others increases the transmission of probable MERS-CoV cases to others [12, 14]. Worldwide awareness of MERS-CoV is low, the disease has high intensity and lethality with unknown mode of transmission and source of MERS-CoV infection (i.e. whether zoonotic or human disease) [15]. Therefore, it is necessary to design and implement a research to identify some unknown epidemiological aspects and also determine the current epidemiological situation of MERS-CoV and its mortality risk factors in order to prevent, control and anticipate effective interventions.

## Methods

Permission was obtained from WHO to conduct this analytical-descriptive epidemiological study. Using census method, data related to laboratory-confirmed MERS-CoV cases between 1.1.2017 to 17.1.2018 were extracted from disease outbreak news on MERS-CoV from WHO website as follows. Demographic information such as age, gender, reporting country, city, health care worker; clinical data and exposure status of MERS-CoV cases including comorbidities, exposure to camels, camel milk consumption, exposure to MERS-CoV cases, day/month of symptom onset, day/month of first hospitalization, day/month of laboratory confirmation, final outcome (dead or survived) of MERS-CoV cases were recorded.

### Statistical analysis

All statistical analyses were conducted using SPSS, version 21 (IBM Inc., Armonk, NY, USA). Quantitative measurement was expressed by medians and qualitative variables were presented as absolute frequency and percentage. Logistic regression was used to calculate the odds ratio (OR) with a 95% confidence interval in order to assess the probable relationship between risk factors and final outcome (dead/survived) of laboratory-confirmed MERS-CoV cases. *P* values of less than 0.05 were regarded as statistically significant.

## Results

A total of 229 MERS-CoV cases, including 70 deaths (30.5%), were recorded in the disease outbreak news on WHO website from 1.1.2017 to 17.1.2018. Based on available details in this research, case fatality rate (CFR), which was calculated by the number of deaths per total number of cases in both genders, was 30.5% (70/229) [32.1% (55/171) for males and 25.8% (15/58) for females].

Overall, the disease occurrence was higher among men [171 cases (74.7%)] in comparison to women [58 cases (25.3%)], as well as in people with comorbidities (151 cases [65.9%] relative to people lacking comorbidities [44 cases (19.2%), in those with exposure to camels [77 cases (33.6%) than those without exposure to camels [26 cases (11.4%)], in individuals consuming camel milk [61 cases (26.6%)] than those not consuming it [28 cases (12.2%)] and also in people who had exposure to MERS-CoV cases [59 cases (25.8%) relative to people not exposed to MERS-CoV cases [24 cases (10.5)].

The median age of subjects was 53.2 years (range: 10–89 years). To assess the effect of several potential risk factors on death in morbid cases related to MERS-CoV infection, we used OR index in order to better understand the mechanism of this relationship, and we reported both crude and adjusted OR. Based on this indicator, variables such as comorbidities and exposure to MERS-CoV cases were significantly associated with mortality in affected people with MERS-CoV infections (Table 1).

**Table 1:** Background data and the effect of various potential risk factors on death related to MERS-CoV infection in morbid cases in the world

Characteristic	Subgroup	Frequency (%)	Number of deaths (%)	Crude OR [95% CI]	Adjusted OR [95% CI]
Age (year)	10-19^a^	8(3.5)	1(1.4)	1.00	1.00
	20–29	18(7.9)	3(4.3)	0.7 [0.06, 8.15]	0.7 [0.06, 8.47]^c^
	30–39	30(13.1)	4(5.7)	0.9 [0.08, 9.68]	0.9 [0.9, 10,24]^c^
	40–49	30(13.1)	3(4.3)	1.2 [0.11, 14.33]	1.3 [0.11, 14.79]^c^
	50–59	54(23.6)	20(28.6)	0.2 [0.02, 2.12]	0.2 [0.02, 2.31]^c^
	60–69	42(18.3)	13(18.6)	0.3 [0.03, 2.86]	0.3 [0.03, 3.10]^c^
	70–79	36(15.7)	20(28.6)	0.1 [0.01, 1.02]	0.1 [0.01, 1.07]^c^
	80–89	11(4.8)	6(8.6)	0.1 [0.01, 1.32]	0.1 [0.01, 1.35]^c^
Sex	Male	171(74.7)	55(78.6)	0.7 [0.37, 1.43]	0.7 [0.38, 1.59]^d^
	Female	58(25.3)	15(21.4)		
Health care worker	Yes	25(10.9)	0(41.7)	0.6 [0.59, 0.72]	0.6 [0.49, 0.88]^e^
	No	204(89.1)	70(34.3)		
Comorbidities^b^	Yes	151(65.9)	63(41.7)	3.7 [1.58, 9.03]	2.2 [1.16, 7.03]^f^
	No	44(19.2)	7(15.9)		
	Not available	34(14.8)	0(0.0)		
Exposure to camels	Yes	77(33.6)	30(39.0)	2.1 [0.76, 5.90]	1.7 [.60, 5.05]^f^
	No	26(11.4)	6(23.1)		
	Not available	126(55.0)	34(27.0)		
Camel milk consumption	Yes	61(26.6)	25(41.0)	2.5 [0.90, 7.18]	2.0 [.68, 6.14]^f^
	No	28(12.2)	6(21.4)		
	Not available	140(61.1)	39(27.9)		
Exposure to MERS-CoV cases	Yes	59(25.8)	6(10.2)	3.3 [1.95, 7.35]	2.3 [1.35, 8.20]^f^
	No	24(10.5)	10(41.7)		
	Not available	146(63.8)	54(37.0)		

^a^ Was chosen as a reference age group

^b^ Diabetes, heart disease, chronic bronchitis and other chronic lung diseases

^c^Adjusted for sex, ^d^adjusted for sex, ^e^adjusted for exposure to camels and exposure to MERS-CoV cases, ^f^adjusted for age and sex

Six countries were affected with MERS during the period of this study. The majority of cases (approximately 93.9%) with highest mortality (98.6%) as well as 100% of female cases have been reported from Saudi Arabia (Table 2).

**Table 2:** Distribution frequency and deaths of MERS-CoV cases by country (as of January 1, 2017 to January 17, 2018)

	Number of cases (*n* = 229)			Number of deaths (*n* = 70)		
Reporting country	Male n (%)	Female n (%)	Total n (%)	Male n (%)	Female n (%)	Total n (%)
Saudi Arabia	157(91.8)	58(100.0)	215(93.9)	54(34.4)	15(25.9)	69(98.6)
UAE^a^	7(4.1)	0(0.0)	7(3.1)	1(1.8)	0(0.0)	1(1.4)
Qatar	3(1.8)	0(0.0)	3(1.3)	0(0.0)	0(0.0)	0(0.0)
Oman	2(1.2)	0(0.0)	2(.9)	0(0.0)	0(0.0)	0(0.0)
Lebanon	1(.6)	0(0.0)	1(.4)	0(0.0)	0(0.0)	0(0.0)
Malaysia	1(.6)	0(0.0)	1(.4)	0(0.0)	0(0.0)	0(0.0)

^a^United Arab Emirates

**Fig. 1:**
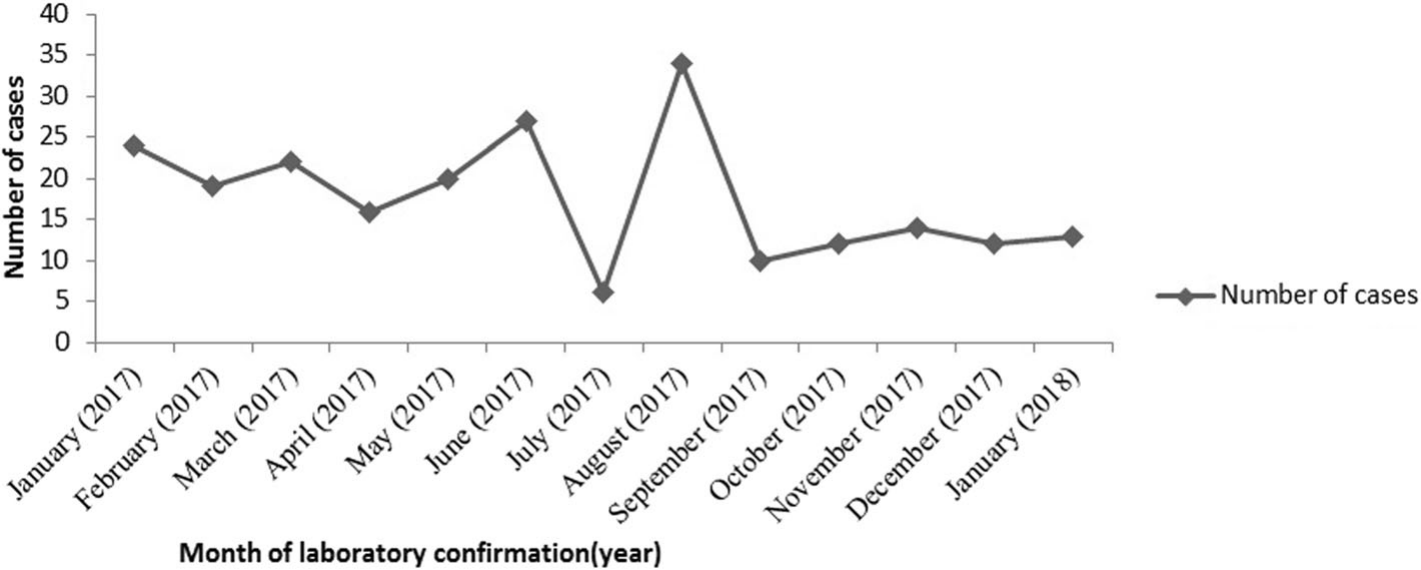
Epidemic curve of confirmed MERS-CoV cases (n = 229) (January 1, 2017, and January 17, 2018)

The epidemic curve of laboratory-confirmed cases of MERS between 1.1.2017 and 17.1.2018 is shown in Fig. 1. It can easily be seen that two peaks are evident in this period: the first at the beginning of April 2017 and the second at the beginning of July 2017. Our results indicate that the number of MERS-CoV cases remained constant from the beginning of September 2017 to the end of January 2018.

**Fig. 2:**
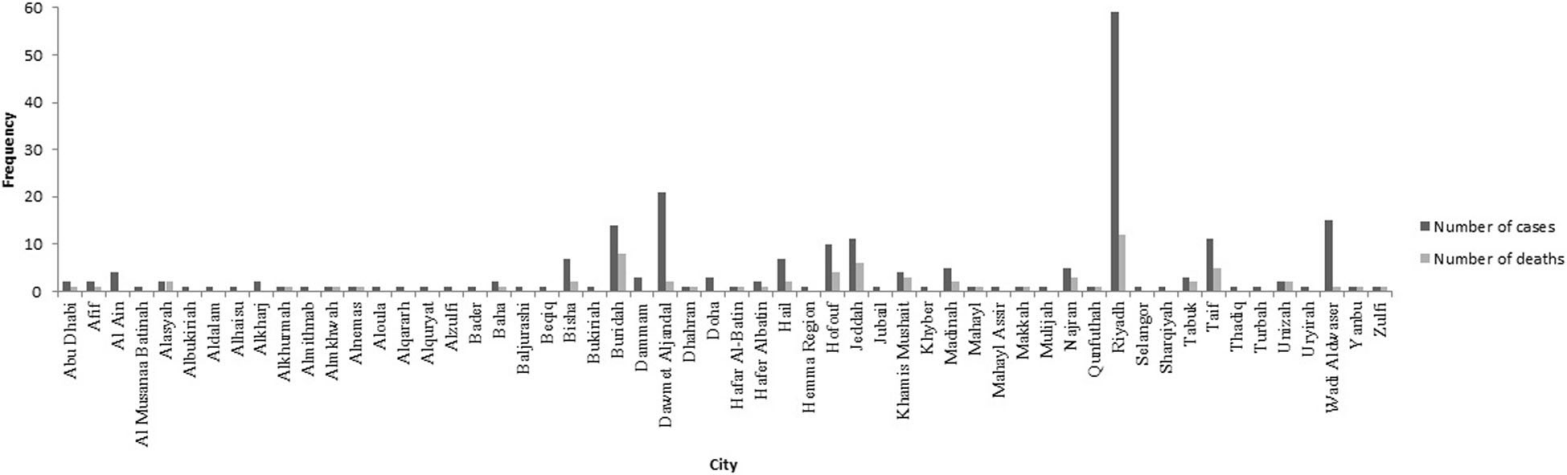
The last distribution of MERS-CoV cases in affected cities in the world (January 1, 2012, and January 17, 2018)

Figure 2 shows that the region of Riyadh, Dawmet Aljandal and Wadi Aldwaser in Saudi Arabia; Al Ain and Abu Dhabi in United Arab Emirates; Sharqiyah and Al Musanaa Batinah in Oman have had the highest occurrence of MERS-CoV infections. This figure also indicates that Riyadh, Buridah and Jeddah in Saudi Arabia had the highest number of deaths in comparison to other cities in different countries. In fact, Saudi Arabia is still the epicenter of this infection worldwide.

## Discussion

The findings have important implications for infection control practice. Especially, we found evidence that was contrary to many studies declaring that the high mortality rates are related to MERS infection with increasing age [16–18]. Our results on MERS-CoV cases in global level showed that all age groups are somewhat at risk of death from this infection. The chance of mortality in MERS-CoV cases in all age groups is fairly equal. Therefore, in the care and treatment of MERS-CoV cases, our results suggest that this important point is better to be considered on behalf of health care staff. In this study, we observed a higher disease occurrence and death of MERS-CoV in men than in women (Table 1). A possible explanation for a higher disease occurrence and mortality of MERS-CoV among men is that men are likely to spend more time outdoors and hence have a higher risk of exposure to a source of infection.

The evidence linking MERS-CoV transmission between camels and humans cannot be ignored. Several studies have shown that persons with direct and indirect contact with dromedary camels had a significantly higher risk of MERS-CoV infection. Our finding was inconsistent with other studies that did not mention such evidence (Table 1). Random error may be one of the reasons for obtaining this result since there were not details of exposure to camels and camel milk consumption for laboratory-confirmed MERS-CoV cases.

Our research is consistent with many studies that provided evidence of human-to-human transmission for MERS-CoV infection [15, 19, 20]. Figure 1 shows two peaks during June until September, which coincides with the largest mass gathering of Muslims around the world in Saudi Arabia to perform Hajj and Umrah ceremony. This finding highlights the effect of congregation in the spread of MERS-CoV infection.

Our findings in Table 2 and Fig. 2 show that most cases are reported from Saudi Arabia after about 7 years since the start of MERS-CoV pandemic (June 2012 to January 17, 2018). So, it seems necessary that epidemiologic investigations are conducted by Ministry of Health in Saudi Arabia and international partners to better understand the transmission patterns of MERS-CoV.

This study had a number of limitations. Assessment of the relationship between mortality related to MERS-CoV infection and some potential risk factor requires reliable sources of mortality data. We used the data recorded in the disease outbreak news on MERS-CoV from WHO website. The quality and accuracy of this data depend primarily on quality of the recorded data reported by national IHR focal point from different countries to WHO. In this study, the researcher was unable to verify the accuracy of the data, which potentially results in information bias. In addition, information for some of the variables was not available and the number of missing data was high, which might introduce a negligible selection bias in results. Another limitation of this research was that possible misclassification of cases may occur due to the respondent’s declarations such as exposure to camels, camel milk consumption, and exposure to MERS-CoV cases, which potentially occurs as a result of measurement bias.

Despite the above limitations, the current analytical-descriptive epidemiological study may have a number of implications for health care policy by using the global data. It also reminds us that effective national and international preparedness plans should be in place as well as measures to prevent, control and predict such viral outbreaks, improve patient management, and ensure global health security.

## Conclusions

The results of this analytical-descriptive epidemiological study revealed and confirmed some potential risk factors for MERS-CoV cases, which were reported as a possible risk factor in previous research studies. In fact, it reminds us that there is probability of MERS-CoV epidemic at any time and in any place without prior notice in today’s “global village”.

## Abbreviations

MERS-CoV: Middle East Respiratory Syndrome Coronavirus
OR: Odds Ratio
UAE: United Arab Emirates
WHO: World Health Organization
